# Stricture of the Urethra III

**Published:** 1915-08

**Authors:** 


**Stricture of the Urethra III.** Henry H. Morton, M. D., Brooklyn, Clinic at L. I. College Hospital.

In making a diagnosis of stricture we cannot tell anything from the history. It simply tells us that the man has an obstruction in his urethra which may be stone, prostate, or stricture. The diagnosis is made by examination, and the flexible bulbous bougie is the best instrument for this purpose. A steel sound is too inexact, as the sound passes through the stricture, gradually dilating it, whereas, a bulbous bougie, on being withdrawn, catches on the stricture band, and a sensation as if passing over a fiddle string is felt.

All soft and recent strictures are treated by dilation and many organized strictures may be treated in this way.

For dilation two instruments are used:—1. Sounds.
2. Dilators.

The effects of dilation are that mechanical stretching and small tears take place in the substance of the stricture, under the mucous membrane, blood supply is increased and absorption is favored.

By repeated dilations the character of the tissue is changed from a live scar, which would contract, to a dead scar, which has little or no tendency to contract.

In all cases begin with sounds, using one large enough to just stretch, the stricture. Massage of the urethra over the sound gives beneficial effects of massage as well as dilation. Sounds should be passed about once a week.

After a dull sized sound no longer dilates the stricture we use a dilator, increasing one or two points at each sitting, followed by an irrigation with silver nitrate 1-4000.

Severe bleeding means too much dilation and patient should have two weeks rest.

False passages are made by pushing the end of sound into the periurethral tissues.

In very tight strictures a filiform guide should be passed and a tunneled sound threaded over it and passed into bladder.

If stricture is so tight no sound can be passed, the guide may be left in for 24 hours causing superficial ulceration, then a flexible bougie is passed and left in for 24 hours. This is continued, using a larger bougie each time till a sound can be passed. This method is not in common use today.

False passages, or in very tight, heavy, dense, tortuous strictures immediate external urethrotomy is indicated.

